# Insect attachment on waxy plant surfaces: the effect of pad contamination by different waxes

**DOI:** 10.3762/bjnano.15.35

**Published:** 2024-04-11

**Authors:** Elena V Gorb, Stanislav N Gorb

**Affiliations:** 1 Department of Functional Morphology and Biomechanics, Zoological Institute, Kiel University, Am Botanischen Garten 9, 24118 Kiel, Germany

**Keywords:** adhesion, *Chrysolina fastuosa*, Chrysomelidae, Coleoptera, epicuticular wax projections, tenent setae, traction force

## Abstract

This study focuses on experimental testing of the contamination hypothesis and examines how the contamination of insect adhesive pads with three-dimensional epicuticular waxes of different plant species contributes to the reduction of insect attachment. We measured traction forces of tethered *Chrysolina fastuosa* male beetles having hairy adhesive pads on nine wax-bearing plant surfaces differing in both shape and dimensions of the wax structures and examined insect adhesive organs after they have contacted waxy substrates. For comparison, we performed the experiments with the same beetle individuals on a clean glass sample just before (gl1) and immediately after (gl2) the test on a plant surface. The tested insects showed a strong reduction of the maximum traction force on all waxy plant surfaces compared to the reference experiment on glass (gl1). After beetles have walked on waxy plant substrates, their adhesive pads were contaminated with wax material, however, to different extents depending on the plant species. The insects demonstrated significantly lower values of both the maximum traction force and the first peak of the traction force and needed significantly longer time to reach the maximum force value in the gl2 test than in the gl1 test. These effects were especially pronounced in cases of the plant surfaces covered with wax projections having higher aspect ratios. The data obtained clearly indicated the impact of waxy plant surfaces on the insect ability to subsequently attach to the clean smooth surface. This effect is caused by the contamination of adhesive pads and experimentally supports the contamination hypothesis.

## Introduction

It has been shown in numerous experimental studies that insects possessing hairy adhesive pads (i.e., specialized tarsal attachment devices) are able to establish a highly reliable contact and adhere successfully to a great variety of substrates having both smooth and microrough topographies [1–3]. However, in cases of waxy plant surfaces, where the plant cuticle is covered by micro/nanoscopic three-dimensional (3D) epicuticular wax projections, insects usually fail to attach to [4–6]. The reducing effect of such plant surfaces on insect adhesion has been shown for many plant and insect species using various experimental approaches, from direct behavioral observations and simple inversion [7] or incline [8] tests up to precise measurements of attachment forces with different experimental techniques, such as pulling [9] and centrifugal [10] setups. It has been demonstrated that not only the presence of wax projections on the plant cuticle surface, but also their size, distribution, and density (number per unit area) influence insect attachment [11–12].

As an explanation for reduced insect adhesion on waxy plant surfaces, several contributing mechanisms have been previously suggested, such as (1) specific micro/nanoroughness created by wax projections (roughness hypothesis), (2) contamination of insect adhesive pads by plant wax during the contact (contamination hypothesis), (3) absorption of the insect pad secretion by the wax coverage (fluid absorption hypothesis), (4) hydroplaning induced by dissolution of the wax in the pad fluid (wax dissolution hypothesis), and (5) detached wax particles forming a separation layer between insect pads and the plant surface and serving as a kind of lubricant (separation layer hypothesis) [7,13].

To date, several experimental studies have been performed to test the first three hypotheses. As for the roughness hypothesis, it was revealed in centrifugal and pulling tests with some insect species bearing hairy attachment pads and mostly artificial substrates having different surface roughness. Insects showed several times higher attachment forces on both smooth and rather coarse microrough surfaces (>3 μm asperity size) compared to force values on 0.3 and 1 μm rough surfaces, where the range of asperity dimensions corresponded to that of typical plant wax projections [1,14–19]. This great reduction in the adhesion force was explained by the strong decrease of the real contact area between the micro/nanorough surface profile and the tips of tenent setae covering insect adhesive pads, which are responsible for establishing an intimate contact with the surface [14].

The fluid absorption hypothesis assumes that because of the high capillarity of the 3D wax coverage, the adhesive fluid may be absorbed from the insect pad surface. The ability to absorb oil, which is one, in beetles possibly even the main, component of the pad secretion [20–22], has been demonstrated experimentally for the wax coverage in the carnivorous plant *Nepenthes alata* Blanco (Nepenthaceae) [23]. Force measurements of the beetle *Coccinella septempunctata* (L.) (Coleoptera, Coccinellidae) on microporous substrates able to absorb both polar (water) and non-polar (oil) fluids clearly showed a strong reduction of the attachment force on these substrates compared to reference smooth solid substrates [24]. The latter result has been explained by absorption of the fluid from insect adhesive pads by porous media and/or the effect of surface roughness. Because of the more elaborate experimental design (three additional force measurements on the solid sample after the test on the porous substrate), a later study with the beetle *Harmonia axyridis* (Pallas) (Coleoptera, Coccinellidae) proved the primary effect of absorption of the insect pad secretion by the porous substrate on the insect attachment force [25].

According to the contamination hypothesis, wax projections can completely or partially detach from the plant surface and adhere to the insect pads covered with the fluid secretion. Such contamination may diminish the attachment ability of the pad. Several previous studies performed with some coleopteran and dipteran species (both having hairy adhesive pads) have reported on grooming behavior of test insects after walking on waxy surfaces of *Eucalyptus niten*s (H. Deane & Maiden) Maiden (Myrtaceae) [26] and *N. alata* [27]. Both earlier and rather recent studies gave direct indications that 3D waxes of the plant species from the genera *Brassica* (Brassicaceae) [8,28–29] and *Nepenthes* [30–33] contaminated insect adhesive pads. Also our previous investigation of twelve waxy plant surfaces verified the contaminating ability of plant waxes, which differed among test plant species depending on the micromorphology, primarily dimensions and shape, of the wax projections [34].

The effect of geometrical parameters of wax projections on their fracture behavior, which in turn determines their contamination ability, was examined using a theoretical mechanical approach [35]. It was demonstrated that during contact formation between insect pads and a plant surface, the wax projections having very high slenderness ratio (i.e., aspect ratio) may easily brake because of buckling, whereas other projections only in some cases fracture by bending.

To date, a very few experimental studies carried out with insects and waxy plant surfaces could confirm only indirectly the contamination hypothesis. Thus, inversion tests performed with the beetle *Chrysolina fastuosa* Scop. (Coleoptera, Chrysomelidae) having hairy adhesive pads on various (among them twelve waxy) plant substrates have shown that *Acer negund*o L. (Aceraceae) stems reduced the further attachment ability of beetles for a certain amount of time, whereas other waxy plant surfaces either did not affect or impaired insect attachment only for a very short period of time [7]. The follow-up study on the contamination of insect pads by plant waxes explained the above effect in a more quantitative way [34].

The aim of this study was to experimentally examine how the contamination of insect adhesive pads by the plant wax contributes to the reduction of insect attachment on waxy plant surfaces and to the subsequent long-term reduction of their attachment ability. We measured the traction forces of *C. fastuosa* male beetles on nine waxy plant surfaces and a reference smooth glass substrate. The experimental design included two force measurements on glass (before and just after experiment on the plant surface) to test whether there is an effect of the plant surface on the ability of insects to subsequently attach to the smooth surface. If there was such an effect, the contamination of pads by the plant wax had a primary effect on the force reduction. Contaminability of insect pads by waxes of different plant species was visualized in an additional experiment.

## Results and Discussion

### Waxy plant surfaces

**Figure 1:**
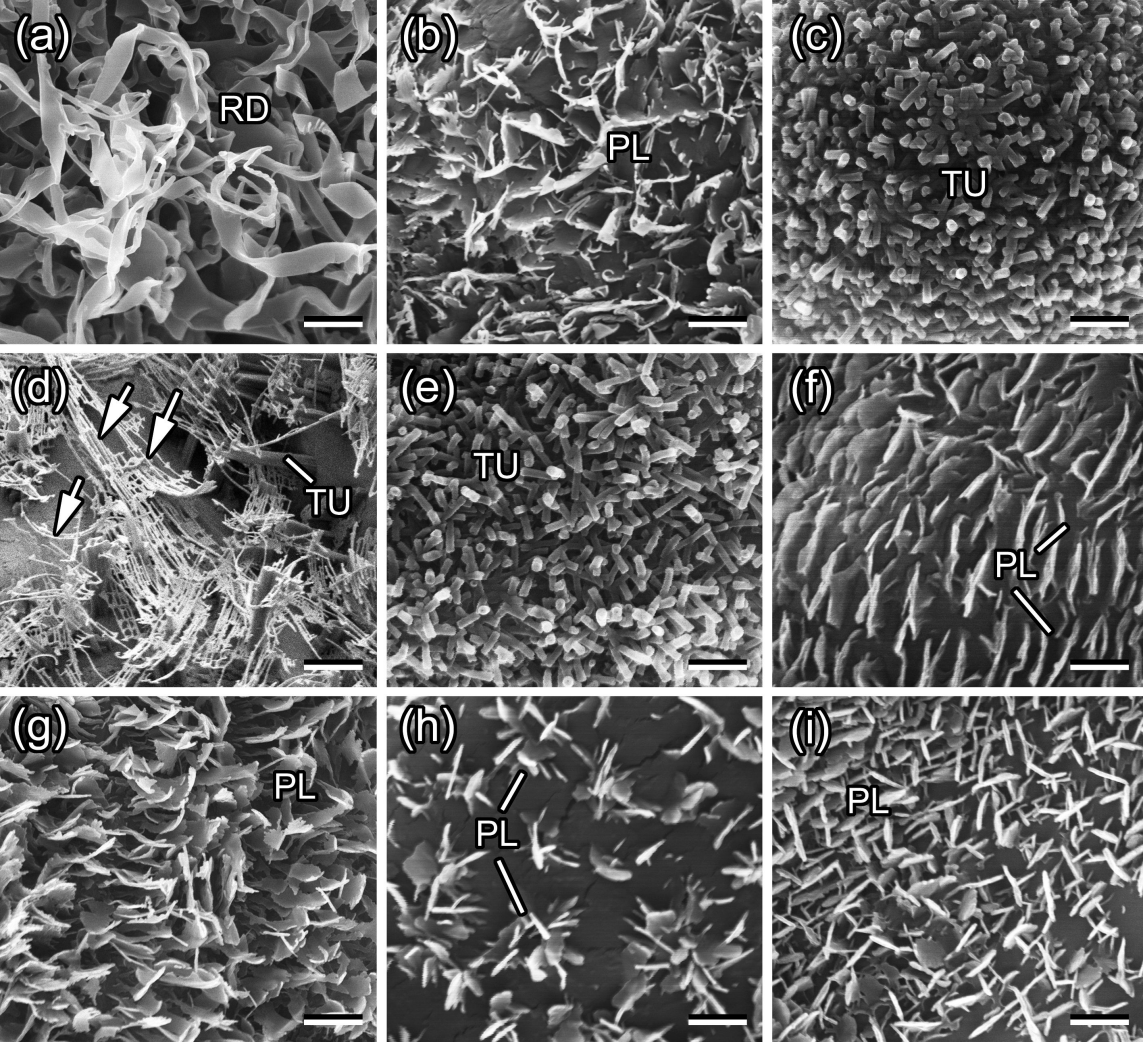
Scanning electron microscopy (SEM) micrographs of waxy plant surfaces in the young stem of *Acer negundo* (a) and adaxial (upper) leaf sides of *Aloe vera* (b), *Aquilegia vulgaris* (c), *Brassica oleracea* (d), *Chelidonium majus* (e), *Chenopodium album* (f), *Iris germanica* (g), *Lactuca serriola* (h), and *Trifolium montanum* (i). PL, wax platelets; RD, wax rodlets; TU, wax tubules. Arrows in (d) denote filament-like branches on top of the tubules. Scale bars: 2 μm (a, b, d, g, h) and 1 μm (c, e, f, i).

The plant surfaces studied are densely covered by different types of epicuticular wax projections depending on the plant species (Figure 1). Both ribbon-shaped polygonal rodlets in *A. negundo* (Figure 1a) and apical filamentous branches of tubules in *B. oleracea* (Figure 1d), although differing greatly in size (length ca. 20 μm in *A. negundo* according to [7,34] and 2 μm in *B. oleracea* according to [19,36]), show very high aspect ratios (ca. 100 [34] and ca. 33 [19,36], respectively). These wax structures have relatively small contact area with the underlying cuticle (*A. negundo*) or with wax tubules (*B. oleracea*). Cylindrical wax tubules in both *A. vulgaris* (Figure 1c) and *C. majus* (Figure 1e) are almost the smallest (<1 μm long [7,34]) structures with the lowest aspect ratios (3–5 [34]) among the plant species studied. As these projections are oriented at various angles in relation to the underlying cuticle, the contact area with the latter also varies. Flat, plate-like membranous (*A. vera*) or irregular (*C. album*, *I. germanica*, *L. serriola*, and *T. montanum*) wax platelets (Figure 1b,f–i), exhibiting intermediate values for both dimension and aspect ratio (0.6–1.7 μm and 9–22, respectively [7,34]), are arranged more or less perpendicularly to the surface. Because of such an arrangement, these platelets could achieve rather firm contact with the underlying cuticle using their whole thin side. Additionally, there are differences in distribution of the wax features. While in *L. serriola*, groups of platelets form clearly distinguishable clusters called rosettes (Figure 1h), the wax projections in other plant species are dispersed rather uniformly and almost completely cover the surfaces.

Data on the wax morphology are in line with our previous studies [7,34] for all plant species except *B. oleracea*, whose projections have been classified as terete rodlets. In later publications [19,36], where cryo-SEM was applied for the examination of plant surfaces, these projections were considered as round or angular tubules with dendrite-like branches on their tops. In the present study, we follow the latter opinion and treat *B. oleracea* wax projections as tubules bearing apical filamentous branches. Data on the dimension and aspect ratio given here for this plant species are related only to the branches, which are usually exposed to the environment, but not to the whole tubules.

### Attachment organs of the *Chrysolina fastuos*a male beetle

#### General morphology

**Figure 2:**
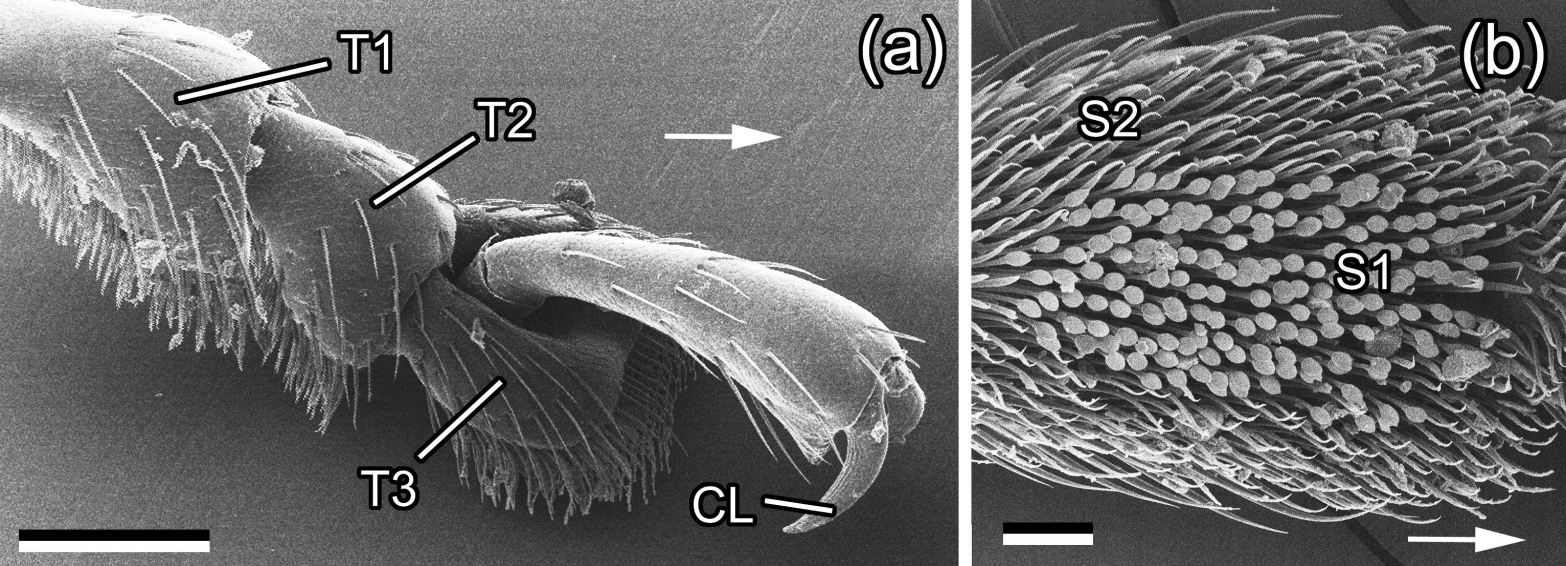
SEM micrographs of attachment organs of a *Chrysolina fastuos*a male beetle. (a) Tarsus with pretarsus, dorso-lateral view. (b) The first (basal) proximal tarsomere (T1), ventral view. CL, claw; S1, setae with discoidal tips; S2, setae with pointed tips; T1-T3, three proximal tarsomeres. Arrows point to the distal direction. Scale bars: 200 μm (a) and 50 μm (b).

The tarsus of *C. fastuos*a possesses two distally located claws and adhesive pads situated on the ventral side of three (out of five) proximal tarsomeres (later referred to as basal, middle, and distal) (Figure 2a,b). In common with most beetles from the family Chrysomelidae [37], this species has hairy tarsal adhesive pads (according to [1,38]). Tenent setae of these pads have different shapes of the tip: (1) a flat discoidal terminal element in mushroom-like setae situated in the central part of the basal and distal tarsomeres (only in males, present in all legs); (2) a flat and widened end plate called spatula in setae located around the field of the mushroom-like setae and in the distal part of the middle pad; and (3) a pointed sharp tip in all setae of the middle pad and in the periphery of the basal and distal pads (Figure 2b).

Recent detailed experimental studies on different beetle species, such as *Leptinotarsa decemlineata* Say, *Gastrophysa viridula* De Geer, *Chrysolina americana* L. (all Chrysomelidae), *C. septempunctata*, and *H. axyridis* (both Coccinellidae) showing a distinct sexual dimorphism in structure and attachment performance of adhesive pads [15,17,24–25,39–42], as well as on mushroom-shaped contact elements of artificial attachment systems [43–44], revealed a strong adaptation of the discoidal tips to long-term adhesion on smooth substrates, especially needed for firm attachment of males to smooth female elytra during mating. Setae with spatula-shaped or pointed tips are better adapted to short-term temporary adhesion and locomotion on various microrough surfaces.

#### Contamination of insect pads by plant wax material

**Figure 3:**
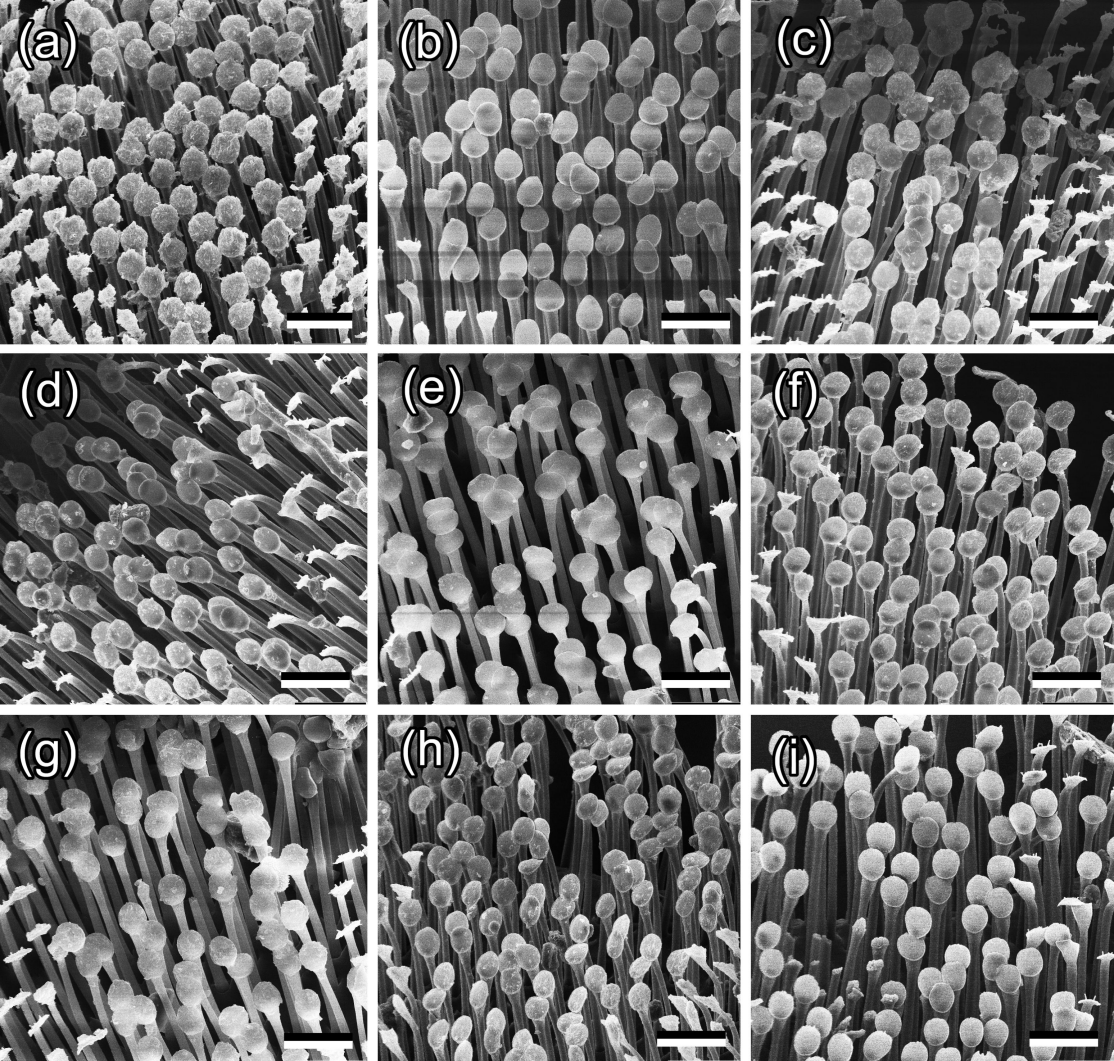
SEM micrographs of the ventral view of the first (basal) proximal tarsomere in *Chrysolina fastuos*a male beetles after they have walked on various plant waxy substrates: *Acer negundo* (a), *Aloe vera* (b), *Aquilegia vulgaris* (c), *Brassica oleracea* (d), *Chelidonium majus* (e), *Chenopodium album* (f), *Iris germanica* (g), *Lactuca serriola* (h), and *Trifolium montanum* (i). Scale bars: 20 μm.

**Figure 4:**
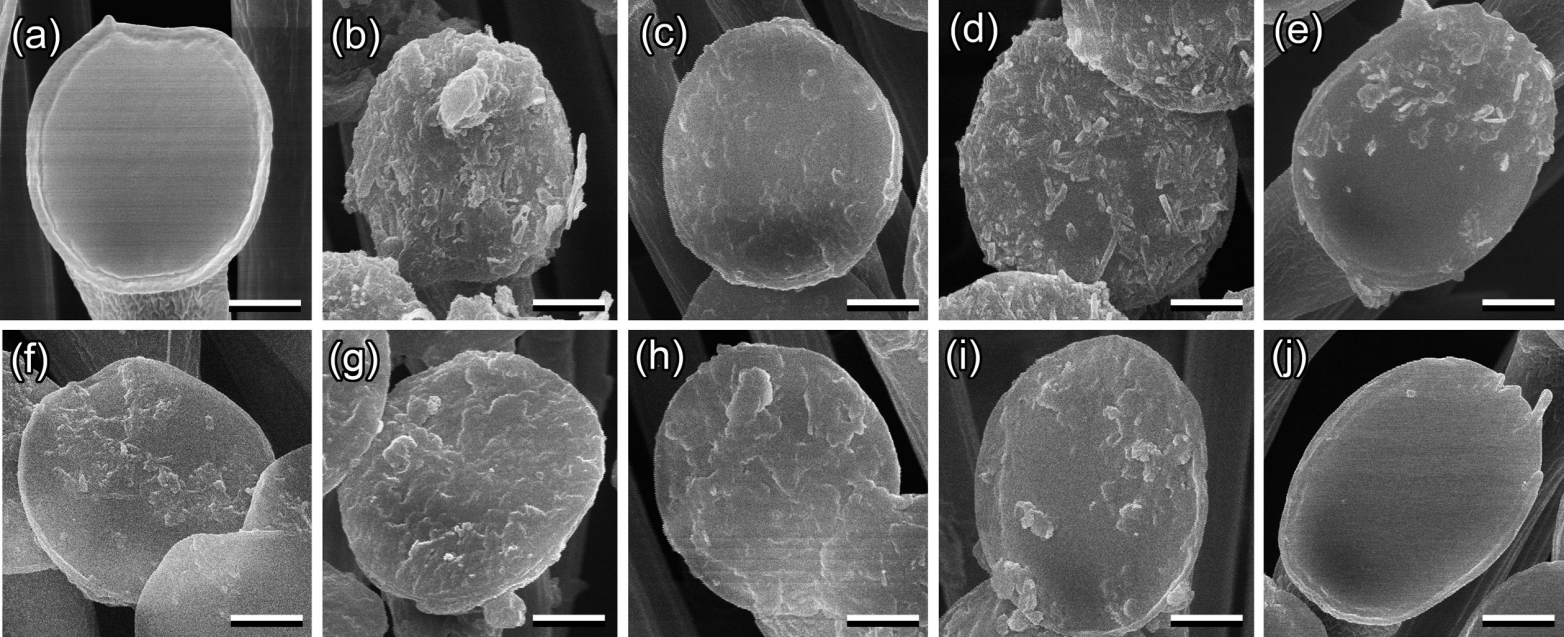
SEM micrographs of the ventral view of discoidal tips in exemplary mushroom-shaped setae of the first (basal) proximal tarsomere of *Chrysolina fastuos*a male beetles in clean (a) and contaminated conditions after the beetles have walked on various plant waxy surfaces: *Acer negundo* (b), *Aloe vera* (c), *Aquilegia vulgaris* (d), *Brassica oleracea* (e), *Chelidonium majus* (f), *Chenopodium album* (g), *Iris germanica* (h), *Lactuca serriola* (i), and *Trifolium montanum* (j). Note differences in the degree of contamination and in the texture of adhered wax depending on the plant species. Scale bars: 2 μm.

As well as in our previous study [34], we considered here only the discoidal setal tips allowing for (1) easier visualization of the contamination and (2) more precise evaluation of the degree of contamination. After insects have walked on various waxy plant substrates, adhesive pads demonstrated contamination of the setal tips by wax material in all cases (Figure 3 and Figure 4). Depending on the plant species, contamination differed in the texture of adhered wax (more or less homogeneous or structured to different extents) and in degree of contamination. Both parameters describing the contamination degree, such as the portion of setal tip surface covered with contaminating wax and the portion of setae contaminated by wax, differed significantly among the plant species used and positively correlated with each other [34]. The degree of pad contamination was higher in the tests with plants having larger dimensions and higher aspect ratios of the wax projections; however, the correlation between these two factors was non-significant in both cases (*P* = 0.068 for dimension and *P* = 0.059 for aspect ratio) [34].

#### Beetle attachment

**Figure 5:**
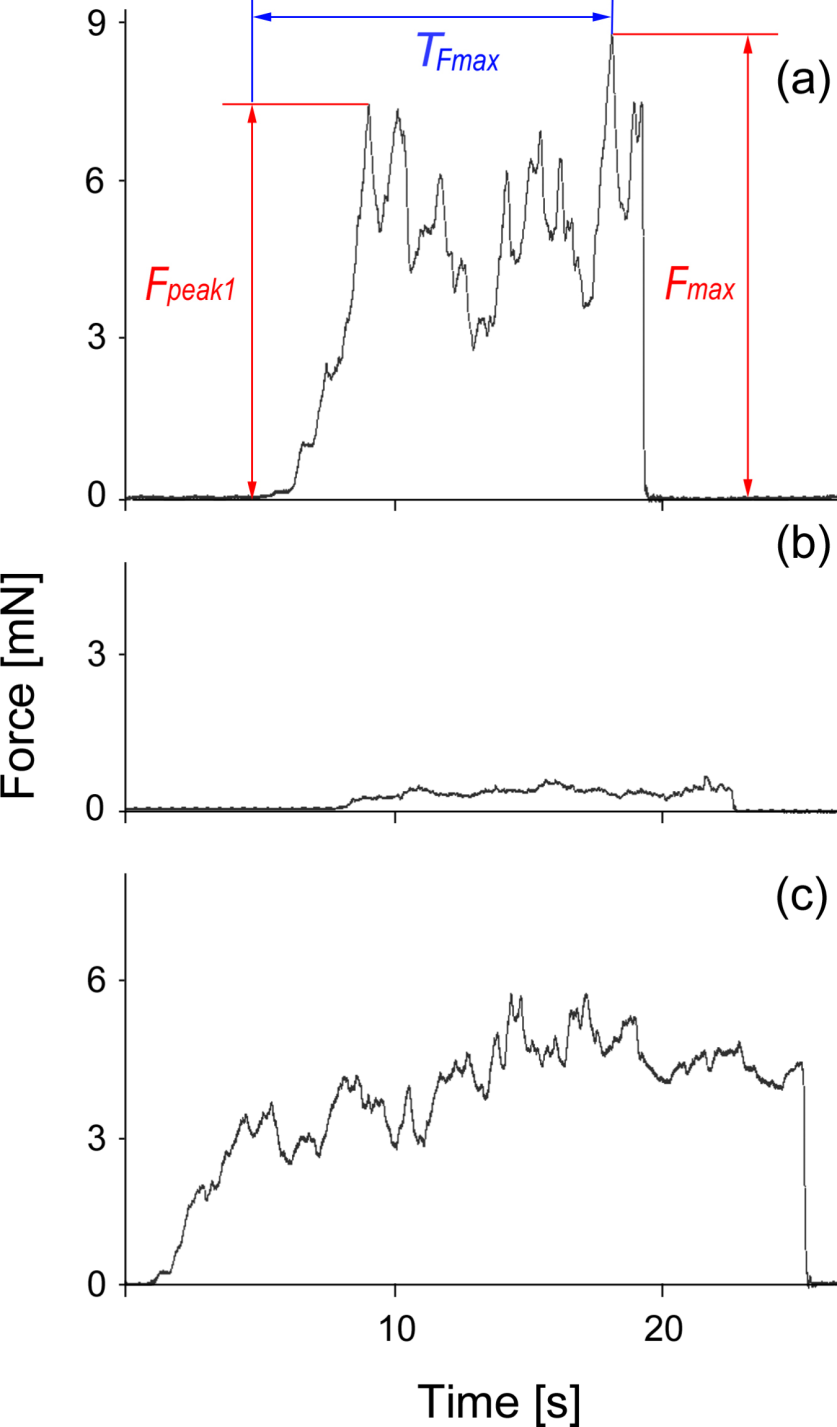
Exemplary force–time curves obtained from one beetle individual in a set of tests on the following surfaces: reference glass gl1 (a), plant (b), and glass gl2 (performed immediately after the test on plant) (c). Here, results for beetle no. 3 tested on an *Acer negundo* waxy stem are presented. *F*_max_, maximal traction force; *F*_peak1_, value of the first peak of the traction force; *T*_Fmax_, time needed to reach the *F*_max_ value.

Figure 5 shows typical force–time curves obtained from one beetle individual in a set of tests on reference glass gl1 (Figure 5a), waxy plant surface (Figure 5b), and in the second experiment on glass gl2 (Figure 5c). Using such curves, the maximal traction force *F*_max_, the value of the first peak of the traction force *F*_peak1_, and the time *T*_Fmax_ needed to reach the maximum traction force value were measured (Figure 5a).

**Figure 6:**
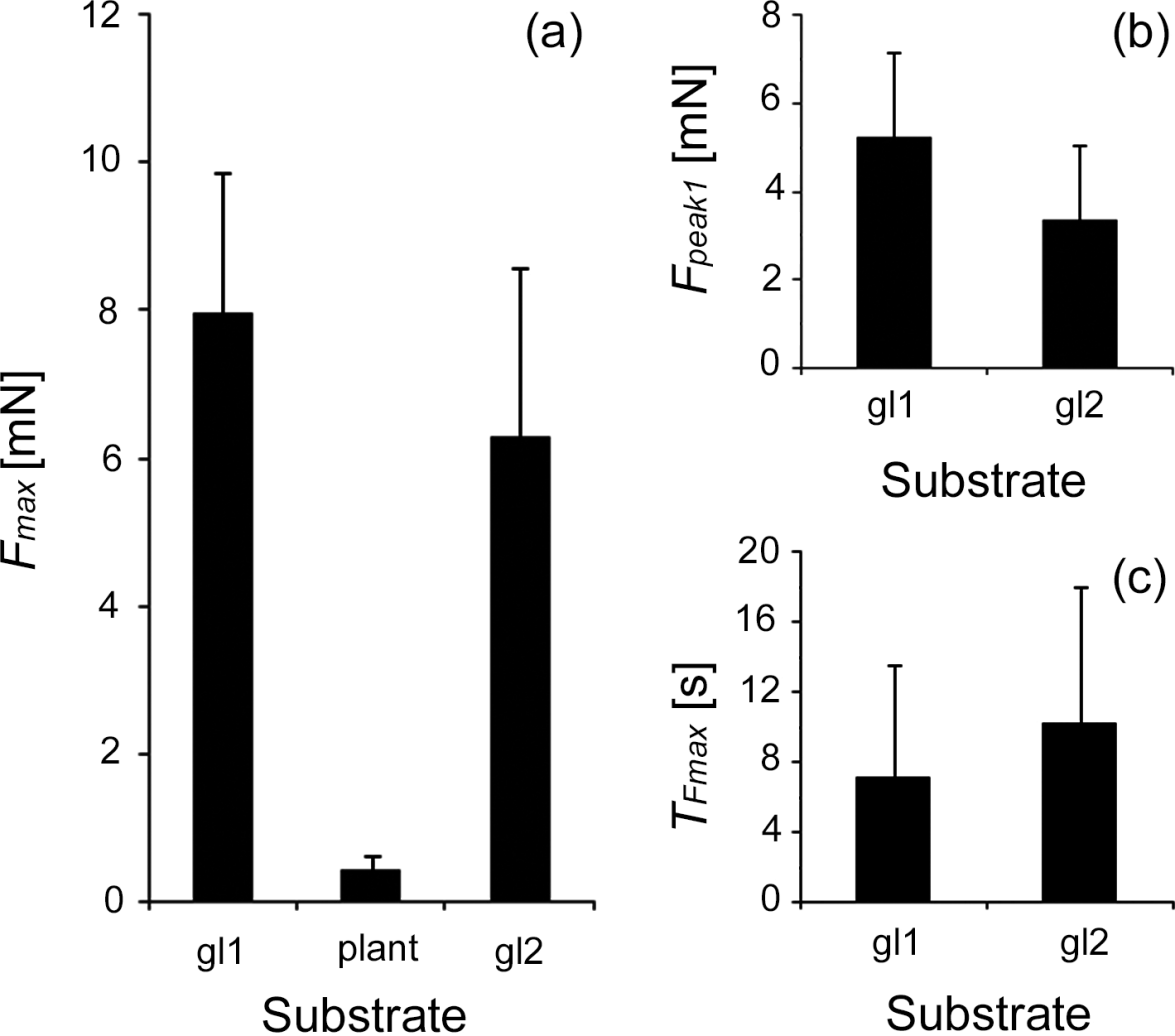
Maximum traction force *F*_max_ (a), first peak of the traction force *F*_peak1_ (b), and time *T*_Fmax_ needed to reach the maximum traction force (c) obtained on waxy plant surfaces and in the first and second experiments on glass. Data on all insects (i.e., from experiments with all plant surfaces) are pooled together. gl1, the first experiment on glass; gl2, the second experiment on glass; plant, waxy plant surfaces.

**Figure 7:**
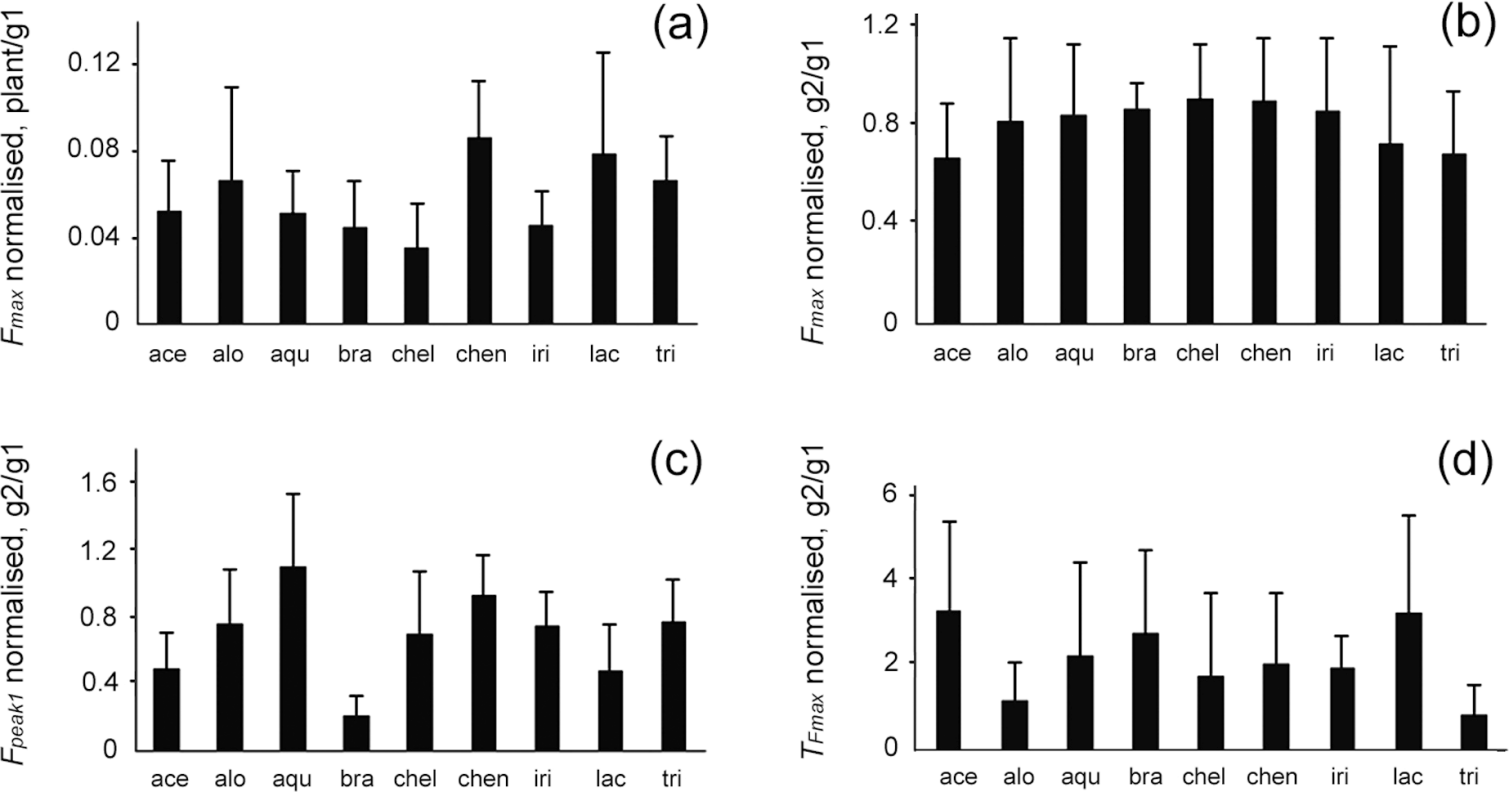
Maximum traction force *F_max_* (a, b), first peak of the traction force *F_peak1_* (c), and time *T_Fmax_* needed to reach the maximum traction force (d) on the waxy plant surface (a) and in the second experiment on glass (b–d) obtained in sets of tests with different plant species. Here, normalized data (divided by the corresponding value obtained in the first experiment on glass) are presented. ace, *Acer negundo*; alo, *Aloe vera*; agu, *Aquilegia vulgaris*; bra, *Brassica oleracea*; chel, *Chelidonium majus*; chen, *Chenopodium album*; gl1, the first experiment on glass; gl2, the second experiment on glass; iri, *Iris germanica*; lac, *Lactuca serriola*; plant, waxy plant surface; tri, *Trifolium montanum*.

Values of *F*_max_, *F*_peak1_, and *T*_Fmax_ were compared among different surfaces inside the experimental set (gl1 vs plant for *F*_max_ and gl1 vs gl2 for *F*_max_, *F*_peak1_, T_Fmax_) for data on all test insects pooled together (i.e., in experiments with all waxy plant surfaces) and for data obtained from five insect individuals on each plant surface (species) separately. Original results on the forces and time in the case of pooled data are presented in Figure 6, whereas for the second case (separate plant species), graphs in Figure 7 show the force and time values normalized to the corresponding ones obtained in the first experiment on glass gl1.

Considering force data obtained from all insect individuals and all waxy plant surfaces tested (pooled data), we found a highly significant reduction (ca. 24-fold in average) of the maximum traction force *F*_max_ on the waxy plant surfaces compared to those obtained in the corresponding first (control) force measurements on the glass substrate gl1 (paired *t*-test: *t* = 26.286, *p* < 0.001) (Figure 6a). The maximum traction forces *F*_max_ from the second experiment on glass gl2 (performed immediately after tests on a waxy plant surface) were significantly lower than those from the first experiment on glass gl1 in all beetles (paired *t*-test: *t* = 5.451, *p* < 0.001) (Figure 6a). Also the comparison of the first peaks of the traction force *F*_peak1_ measured from the force–time curves obtained in the first and second experiment on glass (gl1 vs gl2) showed significantly lower values in the second experiment gl2 (paired *t*-test: *t* = 5.962, *p* = 0.033) (Figure 6b). To reach the maximum traction force values, all insects needed significantly more time during the second experiment on glass gl2 compared with the first experiment on glass gl1 (paired *t*-test: *t* = 2.203, *p* = 0.033) (Figure 6c).

Considering force data obtained in experiments with different plant species, we found that in all plants studied, the waxy surface significantly reduced the maximum traction force *F*_max_ compared to that produced in the first experiment on glass gl1 (Table 1). The force reduction varied greatly between plant species ranging from ca. 12-fold in *C. album* to over 30-fold in *C. majus* (Figure 7a). The comparison of the maximum traction force values *F*_max_ between the first gl1 and second gl2 experiments on glass showed significant differences only in the experiments with *A. negundo, B. oleracea*, and *T. montanum* (Figure 7b and Table 1), where force values were lower in the second experiment on glass g2. The first peak of the traction force *F*_peak1_ was significantly lower in the second gl2 experiment than in the first gl1 experiment on glass in the cases of *A. negundo, B. oleracea*, and *L. serriola* (Figure 7c and Table 1), whereas the difference was not significant in experiments with other plant surfaces. Regarding the time needed to reach the maximum traction force *T*_Fmax_ in the first gl1 and second gl2 experiments on glass, only in the case of *I. germanica*, it was significantly shorter during the second experiment on glass gl2 (Figure 7d and Table 1); for all other plants, this time was not significantly longer.

**Table 1:** Results of the paired *t*-test for comparisons between the first experiment on glass (gl1) and waxy plant surface (plant) and between the first (gl1) and second (gl2) experiments on glass for experimental sets with different plant species.^a^

Plant species	Maximum traction force *F*_max_ gl1 vs plant	Maximum traction force *F*_max_ gl1 vs gl2	First peak force *F*_peak1_ gl1 vs gl2	Time to reach maximum traction force *T*_Fmax_ gl1 vs gl2

*Acer negundo*	*t* = 10.821 *p* = 0.001*	*t* = 3.040 *p* = 0.038*	*t* = 5.305 *p* = 0.037*	*t* = 1.790 *p* = 0.123
*Aloe vera*	*t* = 15.193 *p* = 0.001*	*t* = 1.28 *p* = 0.270	*t* = 1.555 *p* = 0.195	*t* = 0.293 *p* = 0.784
*Aquilegia vulgaris*	*t* = 7.131 *p* = 0.002*	*t* = 1.087 *p* = 0.338	*t* = 0.048 *p* = 0.964	*t* = 1.106 *p* = 0.331
*Brassica oleracea*	*t* = 7.560 *p* = 0.002*	*t* = 2.790 *p* = 0.049*	*t* = 5.305 *p* = 0.006*	*t* = 1.951 *p* = 0.123
*Chelidonium majus*	*t* = 7.907 *p* = 0.001*	*t* = 1.215 *p* = 0.291	*t* = 1.975 *p* = 0.119	*t* = 0.385 *p* = 0.720
*Chenopodium album*	*t* = 10.206 *p* = 0.001*	*t* = 1.139 *p* = 0.318	*t* = 0.987 *p* = 0.380	*t* = 0.648 *p* = 0.553
*Iris germanica*	*t* = 10.746 *p* = 0.001*	*t* = 1.512 *p* = 0.205	*t* = 2.437 *p* = 0.071	*t* = 3.096 *p* = 0.036*
*Lactuca serriola*	*t* = 4.918 *p* = 0.008*	*t* = 2.041 *p* = 0.111	*t* = 3.490 *p* = 0.025*	*t* = 2.279 *p* = 0.085
*Trifolium montanum*	*t* = 10.088 *p* = 0.001*	*t* = 2.824 *p* = 0.048*	*t* = 1.818 *p* = 0.143	*t* = 1.539 *p* = 0.199

^a^*p*, probability value; *t*, test statistics; ^*^, significant difference.

Thus, the comparison of the maximum traction forces *F*_max_ obtained here from *C. fastuosa* males on nine waxy plant surfaces with those measured in the first experiment on the reference glass gl1 demonstrated the anti-adhesive properties of the wax coverage in the studied plant species. This effect was clearly seen when we compared data (maximum traction force values *F*_max_) on all test insects and all plant species pooled together (ca. 24-fold reduction in average) as well as data obtained from five insects tested on each plant surface separately (from 12-fold to over 30-fold reduction). Our results are in line with previously reported findings in many plant and insect species [4–6].

The contaminating ability of plant waxes has been previously shown for many plants [8,28–34]. Our study clearly revealed the effect of pad contamination by plant wax material as an important mechanism of insect attachment reduction on waxy plant surfaces. First, contamination of insect pads by wax was verified for all plant species studied here. Second, we obtained significantly lower values of both the maximum traction force *F*_max_ and the first peak of the traction force *F*_peak1_, and significantly longer times *T*_Fmax_ that the insects needed to reach the maximum traction force value, in the second experiment on glass gl2 compared to the reference (i.e., the first experiment on glass gl1) in all insect individuals and all waxy plant surfaces tested (pooled data). These results show the reduced ability of insects to subsequently attach to a smooth surface after having a previous contact with a waxy plant surface. In combination with our SEM data on contaminated beetle feet, the above outcomes of the force tests indicated that the contamination of pads by the plant wax is responsible for the attachment force reduction on waxy plant surfaces and has a short-term effect on the subsequent attachment to a smooth surface.

The comparison of experimental data among the plant species demonstrated certain differences between the species. Waxy surfaces of *A. negundo* and *B. oleracea* caused a decrease in both force values (maximum traction force *F*_max_ and the first peak of the traction force *F*_peak1_). In these plants, wax projections have highly elongated shapes and exhibit the highest aspect ratios among the plant species studied [19,34,36]. As these wax projections have rather small contact area with the underlying plant surface, they may wholly detach from it and, consequently, easily cause heavy pad contamination. Moreover, according to [35], such wax structures may also readily brake during contact formation with insect pads and contaminate them. Interestingly, it has been previously reported that the *A. negundo* stem surface diminished the further attachment ability of *C. fastuosa* beetles, but the recovery time was relatively short [7]. Also, three other waxy plant surfaces studied here evoked a significant difference between the results of the first gl1 and the second gl2 experiments on glass, however, concerning only one of the attachment parameters measured: *T. montanum* regarding the maximum traction force *F*_max_, *L. serriola* regarding the first peak of the traction force *F*_peak1_, and *I. germanica* regarding the time needed to reach the maximum traction force *T*_Fmax_. Since these plant surfaces are covered by middle-sized wax platelets with intermediate values of aspect ratio [34], they may yield a certain pad contamination, which in turn, may worsen the subsequent attachment ability of beetles for a short time. The waxy plant surfaces bearing small wax projections with low aspect ratio (especially compact, submicroscopic tubules in *A. vulgaris* and *C. majus*) caused inconsiderable pad contamination and, in turn, did not significantly affect further beetle attachment.

## Conclusion

Traction experiments with tethered male individuals of the *Chrysolina fastuosa* beetles equipped with hairy adhesive pads clearly demonstrated a great reduction of attachment (maximum traction) force on all tested nine plant surfaces covered with three dimensional epicuticular waxes. The examination of adhesive pads after they had contacted the waxy plant substrates showed that (1) setal tips were contaminated by wax material and (2) the contamination degree differed between plant species depending on the micromorphology (primarily shape and size/aspect ratio) of the wax projections. The comparison of the maximum traction force value, the first peak of the traction force, and the time needed to reach the maximum force value in experiments on glass performed just before and immediately after the tests on the waxy plant surfaces revealed both significantly lower force values and significantly longer times in the case of the second experiment on glass compared to the first one in all tested insect individuals. When comparing the effect of different plant surfaces, this was more strongly pronounced in *A. negundo* and *B. oleracea* having wax projections with very high aspect ratios. These results evidently demonstrate that the impact of wax-covered plant surfaces on attachment to these surfaces and on subsequent attachment to a smooth surface is strongly influenced by the contamination of insect adhesive pads with the plant wax material.

## Experimental

### Plants

Nine plants species from different plant families were used in the experiments: *A. negundo*, *Aloe vera* (L.) Webb. & Berth. (Asphodelaceae), *Aquilegia vulgaris* L. (Ranunculacear), *Brassica oleracea* L. (Brassicaceae), *Chelidonium majus* L. (Papaveraceae), *Chenopodium album* L. (Chenopodiaceae), *Iris germanica* L. (Iridaceae), *Lactuca serriola* Torner (Asteraceae), and *Trifolium montanum* L. (*Fabaceae*). Young stems (*A. negundo)* or leaves (all other species) of these plants bearing 3D epicuticular wax coverage were collected near Jagotyn (Kyiv District, Ukraine; 50° 15′ 25″ N, 31° 46′ 54″ E) and used fresh in the force tests.

### Insect

The leaf beetle *C. fastuosa* served as a model insect species in this study because it has been used in previous relevant experimental studies on insect attachment to various plant surface types [7] and contaminability of different plant waxes [34]. Additionally, it occurred in great numbers at the study site. The insects were used in the force experiments immediately after capture. In this study, only male beetles (body mass: 26 ± 6 mg, mean ± S.D., *n* = 10) were tested.

### Scanning electron microscopy

To visualize the waxy plant surfaces and attachment devices in the *C. fastuosa* male beetle in both clean and contaminated conditions, scanning electron microscopy was employed. For plant surfaces, small (ca. 1 cm^2^) pieces of plant organs were used. In the case of insect attachment organs, beetles were placed on a clean glass plate and their legs were cut off using a sharp razor blade. To get contaminated insect feet, a beetle was first allowed to walk on a fresh waxy plant surface for 1 min and then immediately transferred to the glass plate with the feet up, avoiding any contact, for cutting off the legs. Air-dried samples (parts of plant organs and clean or contaminated insect legs) were mounted on holders, sputter-coated with gold–palladium (thickness 8 nm for plants and 10 nm for insects), and examined in a Hitachi S-800 scanning electron microscope (Hitachi High-Technologies Corporation, Tokyo, Japan) at an acceleration voltage of 2–20 kV (plants) or 20 kV (insects). In the characterization of the waxy plant surfaces, we used the classification of plant epicuticular waxes according to [45].

### Force measurements

Force experiments were carried out using a load cell force sensor FORT-10 (10 g capacity; World Precision Instruments Inc., Sarasota, FL, USA) connected to a force transducer MP 100 (Biopac Systems Ltd., Santa Barbara, CA, USA) [24,46]. First, in order to make a test beetle incapable of flying, its elytra were glued together with a small drop of molten beeswax. At the same time, a 10–15 cm long human hair was stuck to the wax drop. After the wax had hardened and the insect recovered from the treatment, a free end of the hair was attached to the force sensor. Then, the tethered beetle walked on a horizontally placed test substrate pulling the hair for ca. 30 s, while the friction (traction) force thus produced by the moving insect was registered. Since the insects walked parallel to the measurement axis of the sensor, the recorded force corresponded to the total traction force. Force–time curves obtained were used to estimate the maximal traction force *F*_max_, the value of the first peak of the traction force *F*_peak1_, and the time *T*_Fmax_ needed to reach the maximum traction force value (Figure 5a).

With each insect individual, three successive force tests were carried out on the following substrates: (1) a smooth hydrophilic glass used as a reference substrate (gl1), (2) a waxy plant surface (plant), and (3) once more a glass surface for comparison (gl2). Taking into consideration that these waxy plant surfaces are capable of contaminating insect attachment organs with wax particles [34], we performed the second experiment on glass immediately after the test on the plant, in order to completely exclude a possible effect of feet cleaning or grooming by insects. This aided in the examination of the influence of dirty adhesive pads on the subsequent attachment ability of the beetles. On each set of substrates, five individual male beetles were tested. In all, 135 force experiments were conducted. Force tests were carried out at 22–25 °C temperature and 60%–75% relative humidity.

The statistical analyses of the values of the maximum traction force *F*_max_, the first peak of the traction force *F*_peak1_, and the time *T*_Fmax_ needed to reach the maximum traction force for the comparisons between gl1 and plant and between gl1 and gl2 were performed using the paired *t*-test (SigmaStat 3.5, Systat Software Inc., Point Richmond, CA, USA). The comparisons were conducted for both (1) data on all test insects pooled together, that is, experiments with all waxy plant surfaces (d.f. = 44) and (2) data obtained from five test insects on each plant surface separately (d.f. = 4).

## Data Availability

The data that supports the findings of this study is available from the corresponding author upon reasonable request.
